# Probiotic Supplementation is Associated with Increased Antioxidant Capacity and Copper Chelation in *C. difficile*-Infected Fecal Water

**DOI:** 10.3390/nu11092007

**Published:** 2019-08-26

**Authors:** Mohd Baasir Gaisawat, Michèle M. Iskandar, Chad W. MacPherson, Thomas A. Tompkins, Stan Kubow

**Affiliations:** 1 School of Human Nutrition, McGill University, 21111 Lakeshore, Ste. Anne de Bellevue, Montréal, QC H9X3V9, Canada; 2 Rosell® Institute for Microbiome and Probiotics, 6100 Royalmount Avenue, Montréal, QC H4P 2R2, Canada

**Keywords:** probiotics, *Clostridium difficile*, antioxidant, fecal water, iron chelation, copper chelation, FRAP, DPPH, gastrointestinal model

## Abstract

Probiotic supplementation plays a key role in maintaining intestinal homeostasis due to its ability to modulate gut microbiota. Although their potential as potent antioxidants have previously been explored, their ability to affect the redox status in the gut lumen of healthy subjects or those with gastrointestinal (GI) disorders remains unclear. In our study, we assessed the ability of single strain and multispecies probiotic supplementation to cause a change in the redox status of normal fecal water and in *Clostridium (C.) difficile*-infected fecal water using a simulated gastrointestinal model. Changes in redox status were assessed by ferric-reducing antioxidant power (FRAP), 2’,2’-diphenyl-1-picrylhydrazyl (DPPH), and iron and copper chelation assays. The findings from our study showed that in normal fecal water, probiotic supplements, apart from *Lactobacillus* (*L.) rhamnosus* R0011, showed a significant increase in iron chelation (*p* < 0.05), which was associated with lower FRAP and copper chelation. In *C. difficile*-infected fecal water, all probiotic supplements showed a significant increase in FRAP (*p* < 0.05) and were associated with increased copper chelation. The DPPH assay showed no treatment effect in either fecal water. These findings suggest that *C. difficile* mediates dysregulation of redox status, which is counteracted by probiotics through ferric-reducing ability and copper chelation.

## 1. Introduction

Probiotics have received extensive study as putative therapeutic agents for use toward the prevention and treatment of gastrointestinal (GI) disorders with altered gut microbiota profiles [1,2]. Probiotic supplementation has been well-established in various animal disease models to be associated with decreased intestinal cytotoxic damage, involving downregulation of inflammatory pathways [3,4]. Additionally, probiotics have been indicated to stabilize gut microbiota through different mechanisms such as iron chelation and anti-microbial metabolite production [2], which are associated with antioxidant action. In that regard, bacterial growth cultures have demonstrated that several probiotic strains possess significant in vitro antioxidant activity [5,6,7]. Enhanced plasma antioxidant capacity and decreased oxidative stress biomarkers have also been demonstrated in clinical trials involving probiotic supplementation [5,6,7]. 

The antioxidant potential of probiotic bacteria has been shown to be strain-specific. For this reason, the antioxidant potential is not generalizable to the species level [8]. The strain-specific antioxidant properties of lactic acid bacteria (LAB) are well-documented, with a variety of LAB strains showing antioxidant capacity. However, the extent of their capabilities and the mechanisms by which they act can differ greatly [9]. The ability of probiotics to act as antioxidants is thought to be mediated via several potential mechanisms, i.e., producing antioxidant enzymes such as superoxide dismutase (SOD), glutathione S-transferase, and catalase, as well as by producing metabolites such as lactate [5,6]. In addition, probiotics work through indirect antioxidant mechanisms including reducing bioavailability of metals such as iron, which can also limit pathogen growth through regulating gut microbial profiles [10]. Fecal matter from healthy adults has been shown to possess antioxidant capacity and has been suggested as an important biomarker of gut microbiota homeostasis [11]. A variety of gastrointestinal disorders, such as inflammatory bowel disease and colon cancer, have been linked to an increase in oxidative stress in the gut lumen [12]. On the other hand, antioxidant action has been indicated to play a role in the therapeutic benefits of antioxidants used in the treatment of the gastrointestinal diseases, such as salicylate, butylated hydroxyanisole, and vitamin E [11,13]. Several studies have associated improvements in gut health with the increase in gastrointestinal antioxidant capacity following intake of antioxidants such as polyphenols and tocopherols [14,15]. Moreover, a study by Bianchi et al. (2010) demonstrated that an increase in antioxidant content of feces is associated with an increase in stool bulk, indicating better gut function and health [16]. The potential of probiotic supplements in stabilizing the redox status of human fecal water from healthy subjects or those with GI disorders, however, is unclear.

The antioxidant potential of probiotic LAB could be particularly relevant to *Clostridium (C.) difficile*-mediated infection, as this involves enterotoxin-mediated intestinal toxicity, which is associated with increased free radical production and upregulation of inflammatory pathways [17,18,19]. As LAB strains possess multiple mechanisms of antioxidant action, combinations of these probiotics could increase the capability to regulate redox status changes caused by *C. difficile* infection. To date, *Saccharomyces (S.) boulardii* is the only reported probiotic to show an effective response to the enterotoxins produced by *C. difficile* [20], which could include indirect protective mechanisms involving reduction of free radical generation. There is limited literature on the direct antioxidant properties of *S. boulardii*, but Suryavanshi et al. [21] have shown that this probiotic can act, in growth cultures, as a potent free radical scavenger via production of antioxidant metabolites. Some studies have also shown a reduction in *C. difficile*-associated diarrhea when supplemented with *S. boulardii* and *Lactobacillus (L.) rhamnosus GG* [20]. 

Assessment of antioxidant activity of probiotics has been widely conducted using spectroscopic assays, such as ferric reducing antioxidant power (FRAP), 2,2-diphenyl-1-picrylhydrazyl radical (DPPH), and Trolox-equivalent antioxidant capacity (TEAC) assays [22,23,24,25,26]. Each of these analyses are based on the principle of electron transfer between the oxidant and the antioxidant moieties, i.e., they measure the reduction potential of antioxidants present in a sample. The FRAP and TEAC assays have been shown to have similar redox potentials and are used interchangeably. The DPPH assay is used to determine radical scavenging potential of the sample using a stable synthetic nitrogen-radical [27]. Metal chelation measurements have also been conducted, along with the traditional antioxidant assays, as a potential indication of their mechanism of action [9,28]. 

In the present study, commercially available strains of LAB and *S. boulardii*, as singular treatments and in combination, were used to assess the antioxidative potential of these strains when cultured in a simulated gut digestion model involving either *C. difficile*-infected fecal matter or healthy donor fecal matter. Fecal water (FW) digests were assessed for antioxidant capacity through the FRAP and DPPH assays and for the metal chelation ability of iron and copper. 

## 2. Materials and Methods 

### 2.1. Batch Culture Fermentation

Simulation of GI conditions was done using a dynamic computer-controlled model that utilizes several 250 mL independent fermentation vessels run in parallel. All vessels were maintained under anaerobic conditions by purging with oxygen-free nitrogen gas. Vessel contents were continuously stirred using magnetic stirrers and maintained at 37 °C using heated double-jacketed beakers. The pH monitoring and regulation of each vessel during enzymatic digestion and fermentation was done using python coding of a Raspberri Pi microprocessor (ver. 1B) with an embedded EZO™ pH circuit (Atlas Scientific, NY, USA). Addition of 0.5 M NaOH or 0.5 M HCl was carried out to maintain pH using computer-controlled peristaltic pumps.

One-hundred milliliters of filter-sterilized GI food, previously optimized by Molly et al., [29], composed of 1 g/L of arabinogalactan, 2 g/L of pectin, 1 g/L of xylan, 3 g/L of starch, 0.4 g/L of glucose, 3 g/L of yeast extract, 1 g/L of peptone, 4 g/L of mucin, 0.5 g/L of cysteine, and 40 µL/L of vitamin solution, were added to each vessel (Sigma Aldrich, St. Louis, MO, USA). For batch culture conditions, an adapted method from [30] was used. Oral enzymatic digestion was initiated by adjusting the media to a pH of 7.0 and adding 1.5 mL of ddH_2_O containing 1 g of α-amylase (A3176, Sigma-Aldrich, St. Louis, MO, USA). After 15 min of incubation, the pH of each vessel was decreased to 2.0, followed by addition of 1.5 g of pepsin (P7125, Sigma-Aldrich, St. Louis, MO, USA) in 1.5 mL of 1 M HCl and incubated for 90 min. The vessels were then adjusted to a pH 8.0, followed by addition of 20 mL pancreatic juice (comprised of 12 g/L NaHCO_3_ (Sigma-Aldrich, St. Louis, MO, USA), 6 g/L bile extract (Sigma-Aldrich, St. Louis, MO, USA), and 0.9 g/L pancreatin (Sigma-Aldrich, St. Louis, MO, USA)). After 2 h of pancreatic digestion, 50 mL of fecal slurry were inoculated in each vessel (T = 0 h). Fermentation was carried out for a period of 24 h after inoculation with sampling after every 6 h. Samples taken at each time point were centrifuged at 2000 g for 10 min. The supernatant (hereinafter referred to as fecal water, FW) was filtered using sterile 0.45 µm syringe filters and stored at –20 °C for short-term use and –80 °C for long-term use. All experiments were performed in triplicate.

#### 2.1.1. Fecal Slurry Preparation 

Regular fecal samples were collected from a healthy adult male donor with no history of GI disorders or antibiotic use for a period of at least six months prior. Samples were collected at least three days apart to account for individual variability. Samples were processed within 3 h of collection and frozen at –80 °C in cryoprotectant solution (12.5% glycerol in 0.9% saline (*v*/*v*)) at a ratio of 1:3 *w*/*v*. Prior to inoculation in the gut model, the fecal sample was stabilized at 37 °C overnight under anaerobic conditions. *C. difficile* fecal samples were commercially sourced from BioIVT, USA (male adult; stool positive for toxins A & B). The fecal samples were processed to make a slurry in a similar manner. For each experiment, *C. difficile*-infected fecal samples were prepared by adding *C. difficile* fecal slurry into the regular fecal slurry at a ratio of 1:10 *v*/*v* (5 mL of slurry per vessel). 

#### 2.1.2. Probiotic Treatment Preparation

In this study, eight different probiotic treatments were utilized (five single-strain probiotics and three as combinations). All probiotics were acquired from Rosell® Institute for Microbiome and Probiotics (Montreal, QC, Canada) in powder format and stored at –20 °C until use. The treatments, along with their codes, are as follows: *L. rhamnosus* R0011 (LR11), *L. helveticus* R0052 (LH52), *L. rhamnosus* GG (LGG), *S. boulardii* (SB), *Bifidobacterium (B.) longum* R0175 (BL175), ProtecFlor^TM^ (commercially available combination of LRR, LHR, BLR, and SB) (PROTO), a combination of *L. rhamnosus* R0011, *L. helveticus* R0052 and *B. longum* R0175 (LR + LH + BLR), and a combination of LGG (10 billion cfu) and SB (5 billion cfu) (LGG + SB). Except where specified, all treatments were used at a dose of 1 billion cfu/vessel. All treatments were premixed at room temperature in 2 mL sterile 1× PBS before addition to any vessel (T = 0 h). Two milliliters of 1× PBS were used as negative control (blank).

#### 2.1.3. Experimental Repeats

Each fermentation experiment of an individual treatment, including blank, was performed in triplicate. Samples of each repeat were tested in triplicate for all the assays performed. 

### 2.2. Antioxidant Assays

#### 2.2.1. Chemical Reagents

Chemicals, including 2,2-diphenyl-1-picrylhydrazyl radical (DPPH; S7670), 2,4,6-tris(2-pyridyl)-s-triazine (TPTZ; T1253), ferric chloride (236489), sodium acetate trihydrate (S7670), L-ascorbic acid (A7506), salicylic acid (247588), sodium hydroxide (221465), potassium nitrate (P6083), 4-(2-hydroxyethyl)-1-piperazineethanesulfonic acid (HEPES; 113784), 2,2’,2’’,2’’’-(Ethane-1,2-diyldinitrilo)tetra acetic acid (EDTA) disodium salt dihydrate (ED2SS), L-cysteine (168149), sulphanilamide (S9251), and *N*-1-Naphhthylethylenediamine dihydrochloride (N9125), were obtained from Sigma-Aldrich (St Louis, Mo). Cupric sulphate pentahydrate (CuSO_4_.5H_2_O; BP346-500), methanol, hydrochloric acid, sulfuric acid, and glacial acetic acid were obtained from Fisher Scientific (USA). Trolox ((±)-6-Hydroxy-2,5,7,8-tetramethylchromane-2-carboxylic acid; 218940050), ferrozine (3-(2-pyridyl)-5,6-di(2-furyl)-1,2,4-triazine-5’,5’’-disulfonic acid disodium salt; 410570010), and pyrocatechol violet (3,3’,4-trihydroxyfuchsone-2’’-sulfonic acid; 146540050) were obtained from Acros Organics (Morris, NJ, USA). Water was purified with a MilliQ filtration system. All reagents were of analytical grade purity. Handling of chemicals and discarding of waste were done in accordance with safe lab procedures using a chemical hood and appropriate protective gear. 

#### 2.2.2. Ferric Reducing Antioxidant Power (FRAP) Assay

The FRAP assay was used to measure the total antioxidant capacity of FW based on a 96-well plate adapted method of Benzie et al. (1996) [31]. Briefly, in a 96-well plate, 30 µL dH_2_O and 10 µL of sample or standard were added, followed by 200 µL of pre-incubated FRAP reagent at 37 °C (10:1:1 *v*/*v*/*v* of 300 mM sodium acetate at pH 3.6, 10 mM TPTZ in 40 mM HCl, and 20 mM FeCl_3_.6H_2_O). The contents of each well were mixed for 10 s and absorbance was read at *λ* = 593 nm after 8 min of reaction time using a uQuant microplate reader (BioTek Instruments, Winooski, VT, USA). Seven equally distributed serial dilutions of 1 mM ascorbic acid were used to generate the standard curve (*R*^2^ = 0.999). Results were expressed as mg ascorbic acid equivalents per liter of FW (mg AAE/L). 

#### 2.2.3. 2’,2’-Diphenyl-1-picrylhydrazyl (DPPH) Assay

The DPPH assay was used to assess the radical scavenging capacity of the FW toward the DPPH radical, leading to decolorization of the DPPH methanol solution. The method follows the procedure proposed by [32]. Undiluted aliquots from each experimental run were tested in triplicate. A standard curve was prepared with evenly spaced dilutions of 1 µM Trolox in methanol, with only methanol solvent as blank (*R*^2^ = 0.998). In a 96-well plate, 190 µL of 0.15 mM DPPH-methanol stock reagent solution (pre-measured for a range of optical density value between 0.7–0.9 at 517 nm) was mixed with 10 µL of standard or sample and left to react for 30 min at 25°C. The decrease in absorbance was measured at *λ* = 517 nm using uQuant microplate reader (BioTek Instruments, Winooski, VT, USA). The antioxidant capacity (% of DPPH inhibition) was calculated as [1 − (A _Sample_ / A _Blank_)] × 100. Results were expressed as mg Trolox equivalents per liter (mg TE/L).

#### 2.2.4. Cu (II)+ Chelation Assay

The ability of the FW to chelate Cu^2+^ was measured by the procedure outlined in [33]. Briefly, in a 96-well microplate, 30 µL of sample or standards were pipetted in triplicate, followed by the addition of 200 µL of 50 mM sodium acetate (at pH 6.0) and 30 µL of 100 mg/L CuSO_4_.5H_2_O. After 2 min of reaction time, 8.5 µL of 2 mM pyrocatechol was added. The microplate was placed on a shaker for 10 min, followed by 10 min of rest at 25 °C. Absorbance was read at *λ* = 632 nm and chelating ability was calculated as chelation (%) = [A _Sample_ /A _Control_] × 100. A standard curve was prepared using serial dilutions of 100 mg/L disodium ethylenediamine tetraacetic acid (EDTA.Na_2_) (*R*^2^ = 0.99). Results were expressed as mg EDTA equivalents per liter (mg EDTAE/L).

#### 2.2.5. Fe (II)+ Chelation Assay

The Fe^2+^ chelation capacity of the FW followed the methodology outlined in [33]. Briefly, in a 96-well microplate, 50 µL of sample or standards were pipetted in triplicate, followed by the addition of 160 µL H_2_O (at pH 6.0), 20 µL of 0.3 mM FeSO_4_, and 30 µL of 0.8 mM ferrozine solution. Once mixed, the plates were incubated for 5 min at room temperature. In an additional well, ferrozine solution was replaced with H_2_O for each sample to account for background coloration. A volume of 50 µL of H_2_O alone along with all reagents was used as a control. Absorbance was read at *λ* = 562 nm. The Fe^2+^ chelating ability was calculated as chelation (%) = [(A _Sample_ -A _Solution w/o ferrozine_)/A _Control_] × 100. The standard curve was prepared using serial dilutions of 100 mg/L EDTA.Na_2_ (*R*^2^ = 0.98). Results were expressed as mg EDTAE/L.

### 2.3. Statistical Analysis

All data are reported as means ± standard error of mean (SEM). Data for all assays were analyzed using two-way ANOVA using Probiotic Treatment (nine levels) and Time (five levels) as factors. For multiple comparisons, Tukey’s HSD post hoc test was carried out to compare treatments to control. The means of all time points were jointly considered when no significant interactions in the two-way ANOVA were observed. When significant interactions between time and treatment were observed, the mean of each time point within a treatment was individually compared to its corresponding time point within the control. Correlation analysis was performed using non-parametric Spearman’s rank-order correlation coefficient (ρ-value) to measure the degree of association between each pairwise variable. Statistical significance was set at *p* < 0.05. All statistical analyses and graphs were performed using JMP ® v14.2 (SAS Institute, Cary, NC, USA).

## 3. Results and Discussion

In this study, FRAP and DPPH assays were used to measure the redox status of the fecal water obtained from colonic reactors following simulated upper GI digestion involving probiotic treatments. Iron and copper chelation assays were conducted to assess the capability of the probiotics to affect the chelation ability of the fecal water.

### 3.1. FRAP and DPPH Antioxidant Capacity of Fecal Water

**Figure 1:**
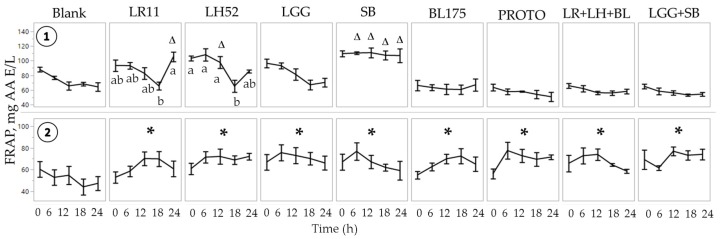
Antioxidant capacity of fecal water (FW) as measured by ferric reducing antioxidant power (FRAP). 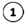 Normal FW 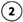 *C. difficile*-infected FW. Values are shown as mean ± SEM. The symbol ∆ represents significant differences (*p* < 0.05) between treatment at a particular time point and blank at the corresponding time point. The symbol * represents significant differences between treatment and blank (*p* < 0.05) when the means of all time points are jointly considered. Means at time points within treatments without a common letter are significantly different (*p* < 0.05). LR11 = *L. rhamnosus* R0011; LH52 = *L. helveticus* R0052; LGG = *L. rhamnosus* GG; SB = *S. boulardii*; BL175 = *B. longum* R0175; PROTO = ProtecFlor^TM^; LR+LH+BL = combination of *L. rhamnosus* R0011, *L. helveticus* R0052 and *B. longum* R0175; LGG+SB = combination of *L. rhamnosus* GG and *S. boulardii*.

Two-way ANOVA results for FRAP showed significant (*p* < 0.05) main effects of treatment and time for both normal and *C. difficile*-infected FW. There was a significant (*p* < 0.05) interaction effect of time and treatment only in normal FW. Therefore, for normal FW, the mean FRAP for each time point within each treatment was compared to its corresponding time point within the blank. Singular probiotics LR11, LH52, and SB showed a significant (*p* < 0.05) increase in FRAP at time 24 h, time 12 h, and time 6 h, 12 h, and 24 h, respectively (Figure 1). No statistical significance was found for LGG and BL175 (*B. longum*-R175) at any given time point, which indicated strain-dependent FRAP radical scavenging activity. Interestingly, a biphasic antioxidant response with time was seen with LR11 and LH52, which showed a significant decrease at 18 h. Although a biphasic response in antioxidant capacity has not been demonstrated with probiotics, it could be speculated that this could be related to binding and release of bacterial exopolysaccharides with gastrointestinal fecal matter at different time points of the digestion processes [34]. Neither of the probiotic combinations appeared to have a significant effect of treatment at a given time point, potentially due to antagonistic effects in the multispecies probiotic combinations. Ranadheera et al. (2014) [35] previously demonstrated that certain probiotic combinations could show a decreased ability to survive in gastrointestinal conditions or adhere to the mucosal layer when compared to their individual probiotics. Furthermore, multispecies combinations could show decreased viability due to competition for essential minerals or secretion of specific bacteriocins [36].

For the *C. difficile*-infected FW, as there was no significant interaction of time and treatment, all time points were jointly considered within each treatment. All probiotic treatments, including the probiotic combinations, showed a significantly (*p* < 0.05) higher FRAP as compared to the control. It is conceivable that the enhanced antioxidant capacity of the combination treatments in the *C. difficile*-infected FW could be related to its cytotoxic characteristics, which led to an upregulation of the reactive oxygen species (ROS) defense mechanisms of the probiotics. Multispecies probiotic combinations have been noted to work in a synergistic manner to maintain redox balance [8]. 

The antioxidant potential of the specific strains used in this study have not been documented previously either in the context of fecal water or fermentation cultures. Similar strains of *Lactobacilli*, *B. longum*, and *S. boulardii*, however, have been associated with antioxidative properties [9,37]. LAB have been shown to produce high levels of glutathione and enzymes such as SOD, along with organic acids such as lactate, which contribute to their overall antioxidant properties [5]. Similarly, *S. boulardii* is thought to produce a range of phenolic metabolites in culture, such as vanillic acid, that contribute to their antioxidative properties [37]. It is thus possible that strains used in the present study possess similar antioxidant mechanisms to increase the reducing ability of FW in both the normal and *C. difficile* FW. 

**Figure 2:**
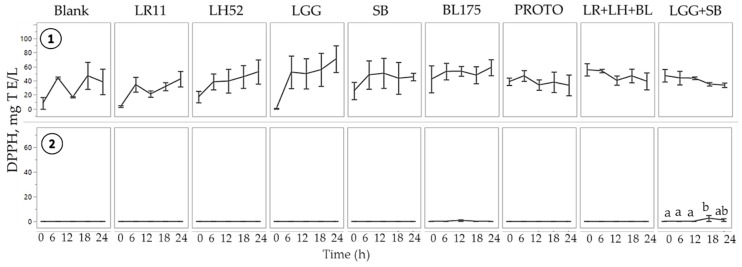
Antioxidant capacity of fecal water (FW) as measured by 2,2-diphenyl-1-picrylhydrazyl radical (DPPH). 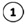 Normal FW 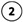 *C. difficile*-infected FW. Values are shown as mean ± SEM. Means at time points within treatments without a common letter are significantly different (*p* < 0.05). LR11= *L. rhamnosus* R0011; LH52 = *L. helveticus* R0052; LGG = *L. rhamnosus* GG; SB = *S. boulardii*; BL175 = *B. longum* R0175; PROTO = ProtecFlor^TM^; LR+LH+BL = combination of *L. rhamnosus* R0011, *L. helveticus* R0052 and *B. longum* R0175; LGG+SB = combination of *L. rhamnosus* GG and *S. boulardii*.

The DPPH assay results showed the potential of normal FW to inhibit or scavenge the DPPH radical, whereas *C. difficile* FW showed no or minimal inhibition across samples (Figure 2). Supplementation with probiotics showed no significant change in DPPH radical scavenging potential in either normal or in *C. difficile*-infected FW. The inability of probiotics to show an effect in the DPPH radical scavenging capacity of either FW could be due to the nature of the DPPH radical and the assay itself. The extremely stable DPPH radical has been shown to have very slow reaction kinetics with some antioxidants as compared to reactions with highly reactive and unstable radicals generated in vivo [27]. Moreover, the assay was conducted using a methanol solvent that might interfere with the reactions that usually happen in aqueous media. From our study findings, it appears that the FRAP assay is a more sensitive indicator for assessing antioxidant reducing ability in FW. The differences in the capacity for inhibition of the DPPH radical between normal FW and *C. difficile*-infected FW could be due to differences in the redox potential of the microbiota itself. In *C. difficile*-infected FW, the absence of DPPH radical inactivation could be due to the increase in ROS production, resulting in a pro-oxidant status [17]. 

### 3.2. Metal Chelation Capability of Fecal Water

Analysis of metal chelation was done to assess the ability of FW to decrease the bioavailability of bivalent metals such as copper and iron. An increase in metal chelation would reduce the available iron and copper used to catalyze free radical polymerization. Studies have previously demonstrated metal chelating potential of certain strains of LAB [9,28,38]. 

#### Determination of Copper Chelation

**Figure 3:**
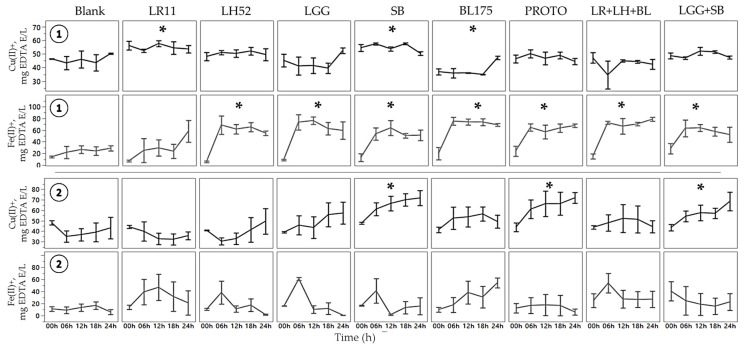
Copper and iron chelation ability of fecal water (FW). 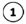 Normal fecal sample; 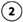 *C. difficile*-infected fecal sample. The symbol * represents significant differences between treatment and blank (*p* < 0.05) when the means of all time points are jointly considered. (LR11 = *L. rhamnosus* R0011; LH52 = *L. helveticus* R0052; LGG = *L. rhamnosus* GG; SB = *S. boulardii*; BL175 = *B. longum* R0175; PROTO = ProtecFlor^TM^; LR+LH+BL = combination of *L. rhamnosus* R0011, *L. helveticus* R0052 and *B. longum* R0175; LGG+SB = combination of *L. rhamnosus* GG and *S. boulardii*).

Two-way ANOVA results for the copper chelation assay showed significant (*p* < 0.05) main effects of treatment for both normal and C. *difficile*-infected FW and no interaction effects. In normal FW, there was a significantly (*p* < 0.05) higher chelation ability of two individual probiotic treatments, *S. boulardii* SB and *L. rhamnosus* LR11 (Figure 3), when compared to blank. Conversely, *B. longum* B175 showed significantly (*p* < 0.05) lower chelating capacity as compared to blank. The probiotic combination treatments (PROTO, LR+LH+BL, LGG+SB) showed no differences in copper chelation relative to the blank despite containing the LR11 and *S. boulardii* SB strains. FW samples with *C. difficile*-infected microbiota showed that only the treatments containing the yeast *S. boulardii* (SB, PROTO, and LLG+SB) showed a significantly (*p* < 0.05) higher chelation capability than blank.

Two-way ANOVA for iron chelation showed a significant effect on treatment only for normal fecal water. There were no interaction effects of time and treatment. Therefore, all of the time points were jointly considered when comparing treatments to blank. Iron chelation results showed a significant (*p* < 0.05) increase in the chelation capability of normal FW supplemented with probiotics, apart from *L. rhamnosus* LR11 when compared to blank. Conversely, the *C. difficile* FW showed no significant increase in the iron chelation capacity of any probiotic supplementation (Figure 3). The results of the probiotic-supplemented normal FW are in concert with previous findings showing that LAB possess metal chelation ability. Lin and Yen et al. (1999) [9] showed that the strain *Streptococcus thermophilus* 821 showed the highest chelating ability for both iron and copper among 19 LAB strains. Six *L. bulgaricus* strains showed high copper chelation and two *B. longum* strains showed a high capacity to chelate both iron and copper. In another study, *L. casei* KCTC 3260 demonstrated the highest iron and copper chelation out of the four LAB strains [28]. Their results also showed that *L. rhamnosus* GG had chelating capacity for both iron and copper and was the only strain to possess significant SOD activity. In another study utilizing LAB, *L. helveticus* CD6 strain showed significant iron chelation ability [38]. 

The results from the present study show the capacity of *S. boulardii* to chelate copper in normal FW, and more importantly, in *C. difficile*-infected FW where it showed significant chelation. Toxins produced by *C. difficile* have been previously linked to enhanced ROS formation, which could lead to metals being used to catalyze oxidation through the Fenton reaction [39]. The ability of *S. boulardii* to reduce ROS formation, as shown by [21], could in part explain its continued capability to chelate copper. The LR11 probiotic, however, was shown in the present study to possess copper chelating ability only in normal FW. Interestingly, all probiotic treatments apart from LR11 showed the capability to chelate iron in normal FW but failed to show any chelation capacity in the presence of the induced *C. difficile* infection. 

### 3.3. Determination of Nitrite, Nitrate, and Protein Carbonyls

Toxins secreted by *C. difficile* have been associated with an upregulation of ROS production in a variety of cell types [17,40]. To assess for compounds generated from ROS reactions, assessments were done for nitric oxide (NO) derivatives and protein oxidation compounds (as outlined in Supplementary Methods S1). 

Nitrite and nitrate quantification were performed in FW as an indirect assessment of NO production. NO, an essential immunomodulatory molecule, is implicated in several pathological conditions when upregulated [41]. In vivo NO is short-lived and rapidly converted to its more stable oxidation end products, nitrite and nitrate. Nitrite quantification in *C. difficile*-infected FW showed low mean quantification values of ~ 0.5 µM in all samples (Supplementary Figure S1). No differences amongst the treatments were observed. Similarly, for nitrate quantification, for each treatment, considering all the time points, mean values of ~ 60 µM were observed. Many values were below the detection limit with a tendency for values to decrease with time for all treatments (Supplementary Figure S2). 

Detection of protein carbonylation is another important method of detection of the extent of oxidative stress. Protein carbonyl groups are stable end-products of protein oxidation, particularly through the formation of aldehyde and ketone groups. These groups could also be formed via oxidative breakdown of the proteins or through lipid peroxidation [42]. In terms of detection of the protein carbonyl moieties in the *C. difficile* FW, only a handful of samples showed carbonyl formation with quantification values of less than 1 nmol/mg protein. The rest of the samples were below the limit of detection of 0.15 nmol/mg protein (Supplementary Figure S3). Low detection of these groups could be due to the formulation of the GI food in the fermentation units that have a low overall protein content (13.3 mg/mL) and a lack of a source of lipids, both of which lead to formation of carbonyl groups. Additionally, it has been stipulated that the critical step in the formation of hydrazones occurs at slightly acidic pH of 3–5 [42]. The pH of fecal water samples was closer to a neutral pH of 6–7, which could have contributed to a lower yield. Overall, the results from nitrite, nitrate, and protein carbonyl quantification showed no apparent increase in oxidation status for these measures in the *C. difficile* FW. 

### 3.4. Correlation of Antioxidant Capacity Assays

The results of antioxidant and metal chelation assays were assessed for correlations to investigate predominant mechanisms of action of probiotics when supplemented in vitro. The data set of each assay (*n* = 15 for each treatment; *n* = 135 in total) was compared using pairwise correlation analysis to assess for any correlations between the antioxidant capacity assays, metal chelation assays and between each of the antioxidant capacity and metal chelation ability assays.

**Figure 4:**
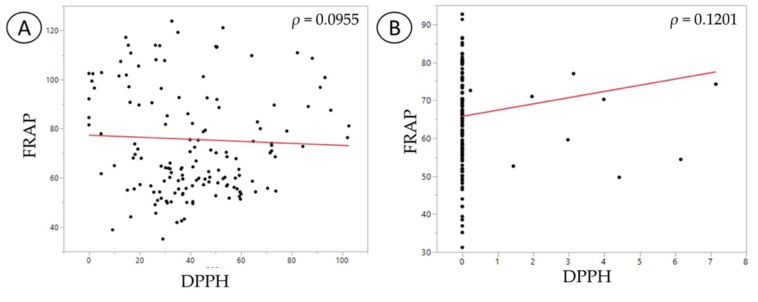
Spearman’s correlation analysis between the antioxidant capacity assays, ferric reducing antioxidant power (FRAP) and 2,2-diphenyl-1-picrylhydrazyl radical (DPPH). (**A**) Normal fecal sample; (**B**) *C. difficile*-infected fecal sample.

The results from the correlation analysis between FRAP and DPPH for each fecal water showed no correlation or trend (Figure 4). The samples of *C. difficile*-infected FW were heavily clustered at the baseline due to the absence of DPPH radical inactivation. 

**Figure 5:**
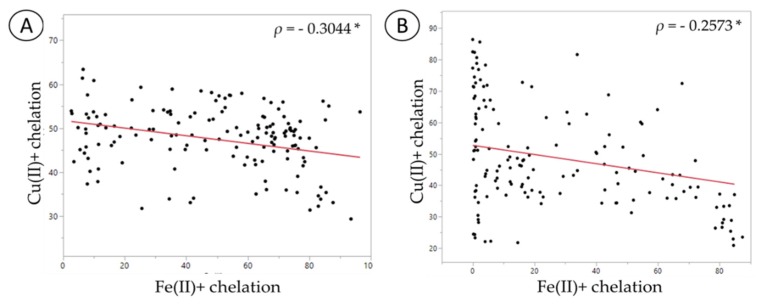
Spearman’s correlation analysis between copper and iron chelation assays: (**A**) Normal fecal sample; (**B**) *C. difficile*-infected fecal sample. The symbol * represents significant correlation (*p* < 0.05).

Comparison of the copper and iron metal chelating ability showed that the copper chelating ability of probiotics was negatively correlated with iron chelation ability in both the normal and *C. difficile*-infected FW (Figure 5; Table 1 and Table 2). These results indicate that an increase of copper chelation in FW was associated with lower iron chelation. 

**Figure 6:**
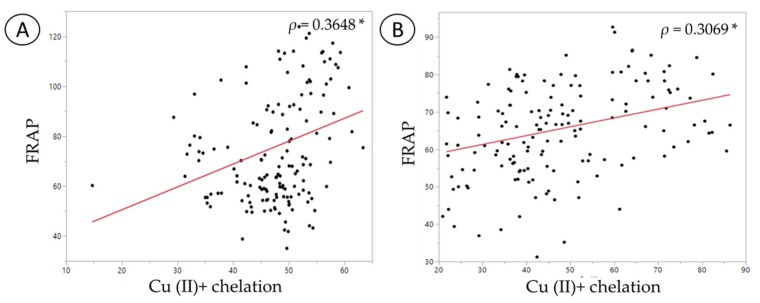
Spearman’s correlation analysis between FRAP and copper chelation ability: (**A**) Normal fecal sample; (**B**) *C. difficile*-infected fecal sample. The symbol * represents significant correlation (*p* < 0.05).

The FRAP assay results showed a strong positive correlation with copper chelation data in each type of fecal water (*ρ* = 0.36 in normal FW and *ρ* = 0.31 in *C. difficile*-infected FW) (Figure 6). Iron chelation showed a strong positive correlation with the DPPH data in the normal fecal sample (*ρ* = 0.41), but no significant correlation was seen in the *C. difficile*-infected fecal sample (Table 1 and Table 2). Likewise, the comparison between FRAP and iron chelation showed a negative correlation in the normal fecal sample (*ρ*= −0.23), whereas no such correlation was observed in the *C. difficile*-infected fecal sample (Supplementary Figure S4). Negative correlation in the normal FW for FRAP and iron chelation could stem from the difference in bacterial products such phenolic metabolites and exopolysaccharides, both of which have been previously demonstrated to possess specificity in transition metal binding [33,43]. Furthermore, no correlations were observed between DPPH and copper chelation in either type of FW (Supplementary Figure S4).

Comparing pairwise correlations in each type of FW showed that FRAP and copper correlated positively (*ρ* > 0.31) and copper and iron chelation correlated negatively (*ρ* < −0.22) in both sets of samples, with both correlations showing statistical significance. The lack of other significant correlations in *C. difficile*-infected fecal samples could have been partly due to the low DPPH values, where almost no inactivation of the DPPH radical was observed. These latter results indicate that the probiotic supplements that show higher reducing ability by FRAP also possess a higher capability to chelate copper. Also, it appears that probiotic supplements that showed an increased copper chelating ability possessed decreased iron chelation ability. The mechanism of action of the apparent affinity for copper could stem from the different profiles of the bacterial secretion product, exopolysaccharides. Bacterial exopolysaccharides have been shown to possess a significant ability to adsorb transition metals such as copper, but they vary greatly in function based on chemical structure and growth conditions, leading to strain specific interactions [43,44].

**Figure 7:**
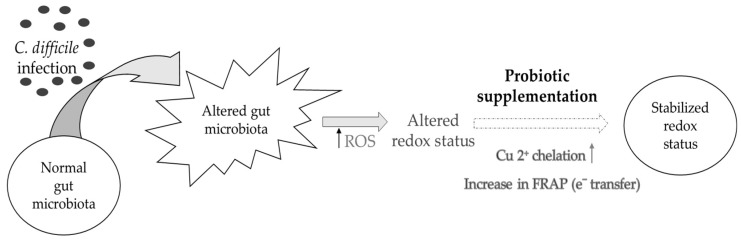
Proposed mechanism of probiotic regulation of redox potential in an altered gut microbiota.

Similar correlations have only been previously performed in studies assessing antioxidant properties of fruit and vegetable extracts. Santos et al. (2017) compared the antioxidant properties of coffee extracts using FRAP, DPPH, total phenolic content (TPC), and iron and copper metal chelation assays [33]. Pairwise correlation analysis conducted on their results showed significant positive correlations between copper chelation and DPPH, FRAP, and TPC. The iron chelation assay showed poor correlations with any of the other assays. This is similar to the present study findings with *C-difficile* FW, as iron chelating capability showed poor correlations with any of the assays. Another study with vegetable juice that underwent simulated digestion showed strong correlations between antioxidant assays FRAP, 2,2’-azino-bis-3-ethylbenzothiazoline-6-sulphonic acid (ABTS), and TPC, but poor correlations of those assays with DPPH [45]. Their results showed significant positive correlations of copper chelation with DPPH, FRAP, and TPC assays. These correlations, however, stem from highly abundant polyphenolic matrices that are absent in our fecal water samples where the antioxidant potential of probiotics was assessed (Figure 7).

## 4. Conclusions

In summary, the present findings have suggested dysregulation in the redox status of the *C. difficile*-infected FW. This phenomenon was indicated by the lack of radical inhibition in the DPPH assay and the reduced chelation capability seen with the iron chelating assay in those samples. Notably, probiotic treatment of the *C. difficile* FW was associated with higher FRAP reducing capacity, which was correlated with a higher capability to chelate copper. As reviewed by Wang et al. (2017), probiotics with antioxidant properties have been associated with protection against some GI diseases, which is linked to inhibition of the adverse effects caused with ROS generated within the GI milieu [6]. With respect to *C. difficile*, the present data supports the concept that probiotic strains with higher reducing and copper chelation abilities could provide an effective strategy to combat the altered redox status associated with *C. difficile* infection (Figure 5). Additionally, these probiotics might help stabilize the altered gut microbiota, an aspect that needs to be explored in more detail in future studies. 

## Figures and Tables

**Table 1:** Spearman’s correlation (*ρ*) of normal fecal samples.

Variables	FRAP	DPPH	Cu (II)+ Chelation	Fe (II)+ Chelation
FRAP	1 *p = n/a*			
DPPH	−0.0645 *p = 0.4572*	1 *p = n/a*		
Cu (II)+ chelation	0.3648 p = 0.0001 *	−0.1497 *p = 0.0831*	1 *p = n/a*	
Fe (II)+ chelation	−0.2289 p = 0.0076 *	0.4149 p < 0.0001 *	−0.3044 p = 0.0116 (*)	1 *p = n/a*

* *p* < 0.05.

**Table 2:** Spearman’s correlation (*ρ*) of *C. difficile*-infected fecal samples.

Variables	FRAP	DPPH	Cu (II)+ chelation	Fe (II)+ chelation
FRAP	1 *p = n/a*			
DPPH	0.1201 *p = 0.1653*	1 *p = n/a*		
Cu (II)+ chelation	0.3069 p = 0.0003 *	0.1187 *p = 0.1704*	1 *p = n/a*	
Fe (II)+ chelation	−0.0162 *p = 0.8516*	0.0822 *p = 0.3429*	−0.2573 p = 0.0026 (*)	1 *p = n/a*

* *p* < 0.05.

## Supplementary Materials

The following are available online at https://www.mdpi.com/2072-6643/11/9/2007/s1, Methods S1.1 Nitrite Determination, Methods S1.2 Nitrate Determination, Methods S1.3 Protein Carbonyl Assay, Figure S1: Nitrite determination in *C. difficile*-infected FW, Figure S2: Nitrate determination in *C. difficile*-infected FW, Figure S3: Protein carbonyl assessment of *C. difficile*-infected FW, Figure S4: Spearman’s correlation analysis of i) FRAP and iron chelation, ii) DPPH and iron chelation, and, iii) DPPH and copper chelation.
