# Supplementary material for: Probiotic Supplementation is Associated with Increased Antioxidant Capacity and Copper Chelation in C. difficile-Infected Fecal Water
Source: Nutrients. 2019 Aug 26;11(9):2007. doi: 10.3390/nu11092007 (PMC6769851; doi:10.3390/nu11092007)
Supplement: Supplementary file 1 [file nutrients-11-02007-s001.zip › nutrients-549561-SI.pdf]

nutrients

MDPI

Article

Probiotic Supplementation is Associated with Increased Antioxidant Capacity and Copper Chelation in *C. difficile*-Infected Fecal Water

Mohd Baasir Gaisawat [1], Michèle M. Iskandar [1], Chad W. MacPherson [2], Thomas A. Tompkins [2] and Stan Kubow [1,*]

Supplementary Methods

S1.1 Nitrite Determination

Nitrite was determined using the sequential-addition Griess reaction in which nitrite reacts with sulfanilamide (SA) in acidic conditions to form a diazonium salt, forming a stable pink azo compound after further reaction with N-naphthyl-ethylenediamine (NED) [37,38]. In this assay, 150 µL of fecal water (FW) was added to 150 µL of 1% (*w*/*v*) SA in 1 M HCl in a 1.5 mL microtube and agitated. One M HCl alone was used to correct for any background nitrite. The mixture is centrifuged at 10,000 g for 10 min at 4 °C. An aliquot of 200 µL of the supernatant was transferred into a new microtube and 100 µL of 0.02% (*w*/*v*) NED in ultrapure water was added. After 15 min of reaction at room temperature, 200 µL was transferred into a standard 96-well assay plate and absorbance was measured at λ = 540 nm. A standard curve was prepared using equally-spaced serial dilutions of 250 µM potassium nitrite (KNO_2) (R^2 = 0.99).

S1.2 Nitrate Determination

Nitrate was measured using a microplate-adapted method of Cataldo *et al.* (1975) [39]. Briefly, salicylic acid underwent nitration by dissolved nitrate under acidic conditions. The transient compound formed a yellow complex when further mixed with an alkaline solution. For the assay, in a 1.5 mL microtube, 10 µL of FW sample was added to 40 µL of 0.05% (*w*/*v*) salicylic acid in concentrated sulfuric acid and vortexed. To determine the nonspecific background concentration, 40 µL was used. After 20 min of reaction at 25 ˚C, 1 mL of 8% (*w*/*v*) NaOH in ultrapure water was added to each tube. A 200 µL aliquot from each tube was added into a 96-well microplate and absorbance was read at λ = 410 nm. Stock nitrate standard solutions (8 mM) were prepared by dissolving 0.81 g oven-dried (100 °C, 1 h) KNO_3 in 1 L ultrapure water. A standard curve was prepared using serial dilutions of 8 mM KNO_3 (R^2 = 0.99).

S1.3 Protein Carbonyl Assay

Detection of protein carbonyl groups was done using the kit provided by Abcam (Cambridge, USA) with the catalogue no. ab126287. Procedures were followed as per manufacturer’s instruction. This kit utilizes 2,4-dinitrophenylhydrazine (DNPH) to derivatize protein carbonyl groups, forming DNP hydrazones which are then quantified spectrophotometrically at λ = 375 nm. The results were expressed as nmol carbonyl/mg protein.

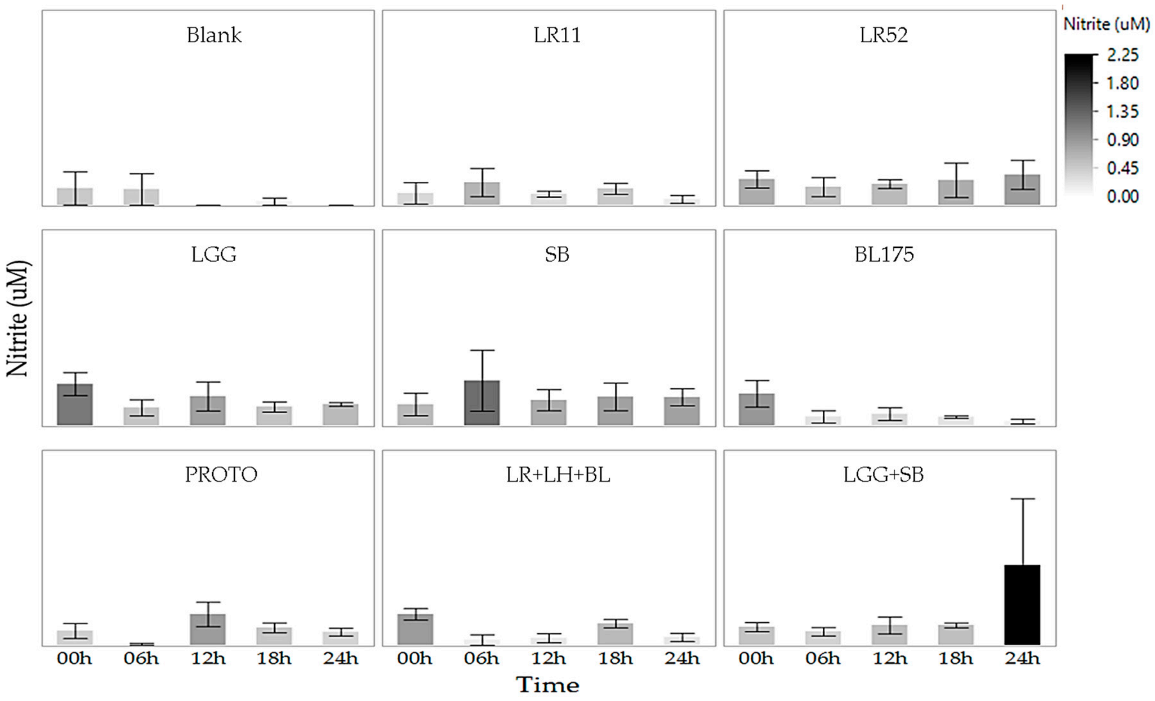

Supplementary Figure S1. Nitrite determination in C. difficile-infected fecal water (FW).

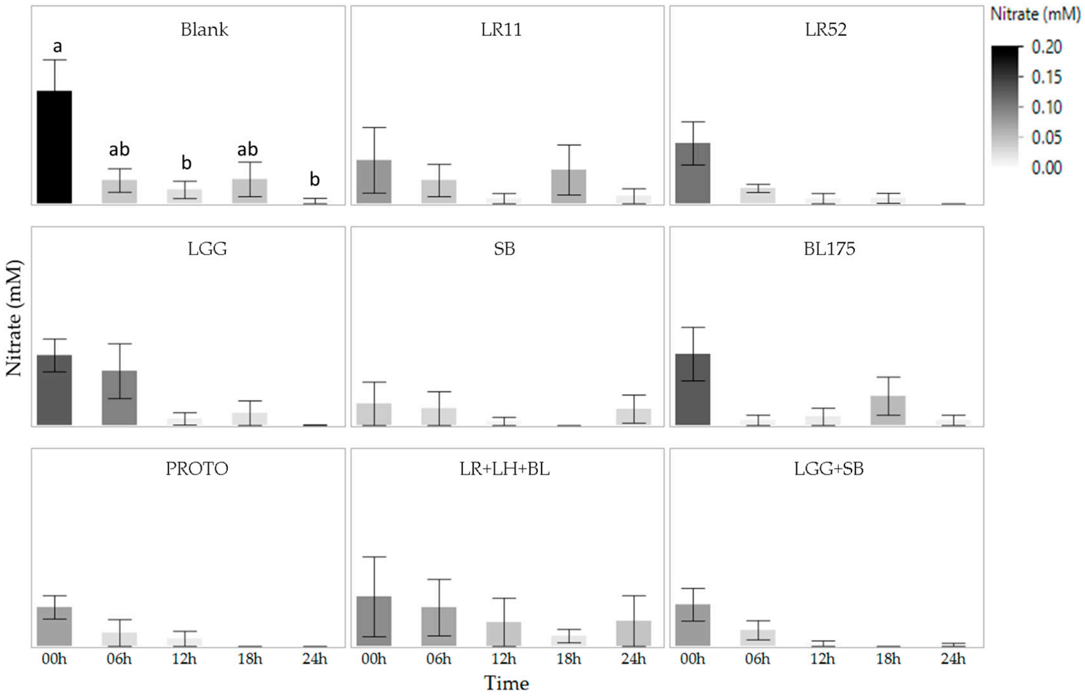

Supplementary Figure S2. Nitrate determination in *C. difficile*-infected FW.

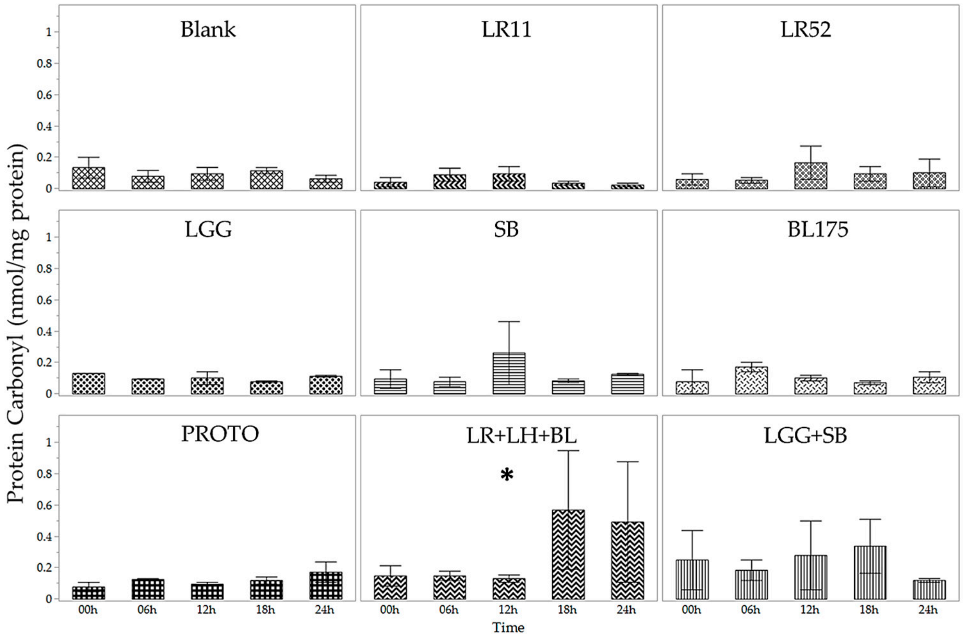

Supplementary Figure S3. Protein carbonyl assessment of *C. difficile*-infected FW. The symbol * represents significant differences between treatments when compared to blank.

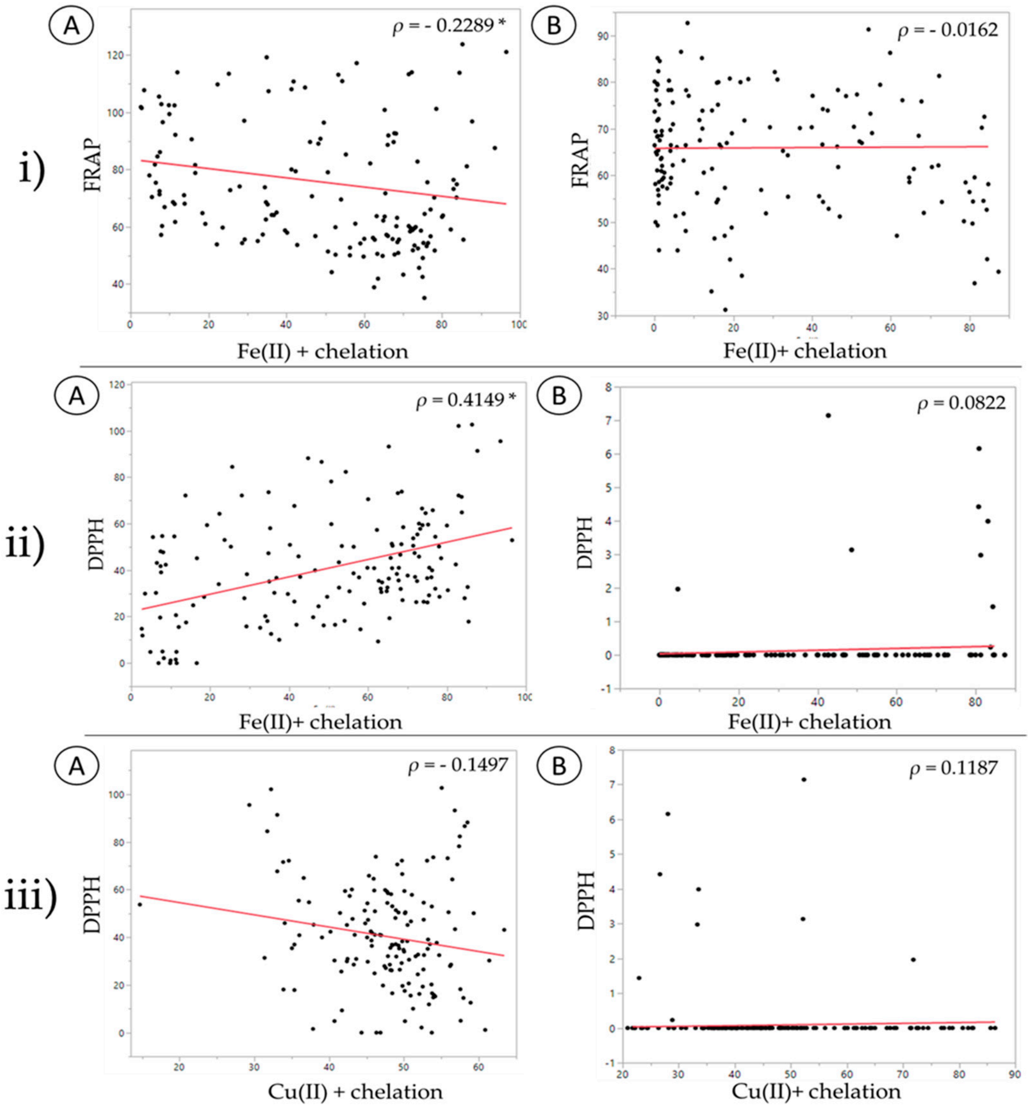

Supplementary Figure S4. Spearman's correlation analysis of i) FRAP and iron chelation ii) DPPH and iron chelation and iii) DPPH and copper chelation. **A**) normal fecal sample; **B**) *C. difficile*-infected fecal sample. The symbol * represents significant correlations ($p < 0.05$).

1. Hachiya, T.; Okamoto, Y. Simple spectroscopic determination of nitrate, nitrite, and ammonium in Arabidopsis thaliana. *Plant Cell Physiol.* **2016**, *7*, 1-8.
2. Moshage, H.; Kok, B.; Huizenga, J.R.; Jansen, P.L. Nitrite and nitrate determinations in plasma: A critical evaluation. *Clin. Chem.* **1995**, *41*, 892–896.
3. Cataldo, D.A.; Maroon, M.; Schrader, L.E.; Youngs, V.L. Rapid colorimetric determination of nitrate in plant tissue by nitration of salicylic acid 1. *Commun. Soil Sci. Plant Anal.* **1975**, *6*, 71–80.

© 2019 by the authors. Submitted for possible open access publication under the terms and conditions of the Creative Commons Attribution (CC BY) license (http://creativecommons.org/licenses/by/4.0/).
